# Understanding breast cancer stem cell heterogeneity: time to move on to a new research paradigm

**DOI:** 10.1186/1741-7015-11-169

**Published:** 2013-07-23

**Authors:** Ferdinando Mannello

**Affiliations:** 1 Department of Biomolecular Sciences, Section of Clinical Biochemistry and Cell Biology, University ‘Carlo Bo’, Via O. Ubaldini 7, Urbino, PU, 61029, Italy

**Keywords:** Biomarkers, Breast cancer, Cancer heterogeneity, CD44/CD24/CD49f, Ep-CAM, Mammosphere, Stem cells

## Abstract

Human breast cancer (BC) is one of the leading causes of death for women worldwide, and is characterized by a group of highly heterogeneous lesions. The morphological and biomolecular heterogeneity of BC cells, accompanied by dynamic plasticity of the BC microenvironment and the presence of stem-like cells, make tumor categorization an urgent and demanding task.

The major limitations in BC research include the high flexibility rate of breast cancer stem cells (BCSCs) and the difficulty of their identification. Improved profiling methods and extensive characterization of BCSCs were recently presented in *BMC Cancer*, highlighting that the majority of BC cells had a luminal EpCAM^high^/CD49f^+^ phenotype, and identification of CD44^high^/CD24^low^ subpopulation of cancer stem cells significantly improves the flow cytometry measurement of BCSCs with higher stem/progenitor ability.

Future developments in single-cell omics will potentially revolutionize cancer biology and clinical practice, providing better understanding of BC heterogeneity, how BCSCs evolve, and which BC cells to target to avoid drug resistance.

Please see related research published in BMC Cancer: http://www.biomedcentral.com/1471-2407/13/289/abstract

## Background

Human breast cancer (BC) represents a group of highly heterogeneous lesions consisting of morphologically distinct subtypes [1], with different molecular/biochemical signatures [2], both between and within tumors [3]. BC is one of the leading causes of death for women worldwide, and also has the second highest morbidity rate worldwide [4]. Although the increased rates of diagnosis of early stage disease in recent years has led to a significantly decreased trend in mortality rate, invasive and hormone-independent BC carries still a bad prognosis and still fairly limited therapeutic options, thus there is an urgent need to improve our understanding the biomolecular basis of BC.

The very high rate of heterogeneity in BC cell phenotypes [5], accompanied by the dynamic plasticity of the breast cancer microenvironment [6,7], make tumor categorization a demanding task, especially in relation to therapeutic responses and risk of disease progression [8]. The only established reason behind this is the underlying presence of a small population of stem-like cells called breast cancer stem cells (BCSCs) [9], which are endowed with the capacity for self-renewal and multi-lineage differentiation, tumorigenicity, and chemotherapy and radiotherapy resistance, features that are responsible for tumor progression, disease recurrence, and metastasis [10].

During the past decades, there have been considerable improvements in isolating and enriching BCSCs, uncovering cellular/tissue biomolecular alterations (through mutation screening, gene expression, microRNA, and proteomic-metabolomic-degradomic profiling). Although the relevant biological role of the breast microenvironment and the cross-talk between epithelial, stromal, and stem cells has been widely and continuously analyzed, the heterogeneity in BC is still not completely understood, which represents a major obstacle to effective cancer treatment and personalized medicine [3,8].

### Breast cancer and stem cell heterogeneity

The clonal expansion and adaptation of BC cells to changing microenvironments [6], and the acquisition of genetic and epigenetic alterations by these cells [11] are well-known dynamic processes contributing to the generation of intra-tumor heterogeneity [12]. In particular, BC heterogeneity can arise from the differentiation of stem-like cells along with the clonal selection that occurs during BC progression, and such heterogeneity represents a major challenge for the design of effective therapies. To make inferences about BC progression, it is important to understand the stem cell origins of the inter-tumor and intra-tumor heterogeneity, which requires more effective BCSC biomarkers.

Two main initial theories have provided some mechanism(s) accounting for BC heterogeneity: 1) the theory of cancer stem cells (CSCs), which suggests that different tumors result from different stem cells, and that all cells within a given tumor are capable of progressing to a higher degree of malignancy [13]; and 2) the theory of clonal evolution, which hypothesizes that different tumors originate from evolution of a single stem cell, and that only the most aggressive clone progresses [14]. A recent study showed that cancer progenitor cells have the capacity to dedifferentiate and acquire a stem cell-like phenotype, indicating that CSC and relatively differentiated progenitors coexist in dynamic equilibrium and are subject to bidirectional conversion [15].

The dominant role of the tumor microenvironment in determining the CSC phenotype characteristics within a malignancy is noteworthy, as it suggests that tumors contain large populations of tumorigenic and non-tumorigenic tumor cells, whose distribution may vary over time [6,16]. Like normal stem cells, CSCs also display biologically significant phenotypic and functional heterogeneity, and their progeny can show diverse plasticity [17], All these tumor cells need to be therapeutically targeted to improve the cure rate in patients with cancer [8].

Recently, outstanding challenges in identifying CSCs, their dependency on a supportive niche, and their role in metastasis have been addressed by a fluid model [16], in which the quality of stemness, rather than being fixed entity, is a flexible quality of tumor cells that can be lost and gained [18].

The major problems/limitations in BC research are represented by the high flexibility of the CSC system (dictated by the microenvironment) and the difficulty of CSC identification (imposed by the current imperfect biomarkers). Although an extensive compilation of molecular CSC markers for distinct human solid tumor types has been reviewed [18], actually none of the known markers are specific for CSC, and only new cell surface marker combinations may improve and hamper reliability, identification, and enrichment of CSCs, thus new biomarker panels are then urgently needed to recognize and quantify more efficiently both circulating and resident BC CSCs.

It has been previously shown that human BCSCs can be isolated and analyzed based on CD44^high^, CD24^low^ and high aldehyde dehydrogenase (ALDH) activity [19,20]. Improved profiling and extensive characterization of BCSCs was presented in *BMC Cancer* by Ghebeh *et al.,* showing the importance of a new biomarker combination for understanding BC carcinogenesis and heterogeneity [21].

### A new and useful BC stem/progenitor marker combination on the horizon

The absence of reliable CSC biomarkers continuously stimulates BC research, in order to identify BCSC in *ex vivo* models, and thus improve their identification and enrichment in the tumor microenvironment [6], and elucidate the biological basis of BC heterogeneity and drug resistance [22].

**Figure 1:**
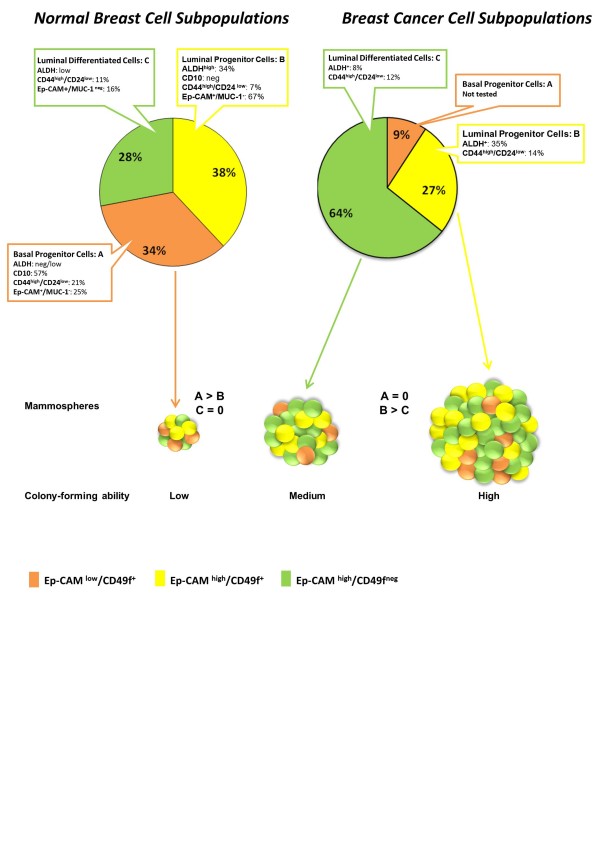
**Schematic representation of differences between normal and malignant breast epithelial stem/progenitor subpopulations.** Comparison of pie charts summarizing the differences and similarities between different epithelial cells within Ep-CAM/CD49f subpopulations, which were presented as subfractions based on stem/progenitor cell markers, in accordance with the data recently described in *BMC Cancer* [21]. The three epithelial cell populations of the normal breast (named A, B and C) are compared with their malignant counterparts, highlighting the peculiarity of each subpopulation. The schematic size of the mammosphere relates to the measured ability of mammosphere/colony-forming cells. Basal progenitor cells showed higher mammosphere colony-forming ability compared with luminal progenitor cells in normal breast cells (A>B, C = 0), whereas in BC, the luminal progenitor subpopulation showed increased ability to form mammospheres compared with differentiated luminal cells. Subpopulations: orange, Ep-CAM^low^/CD49f^+^; yellow, Ep-CAM^high^/CD49f^+^; green, Ep-CAM^high^/CD49f^-^.

To better characterize human normal and malignant breast epithelial cell subpopulations, Ghebeh *et al.*, in a research article in *BMC Cancer*, analyzed a wide panel of breast epithelial stem/progenitor/cancer stem cell markers in normal and malignant breast tissues and BC cell lines, studying subpopulations of cells for mammosphere-forming and colony-forming capacity [21]. These skilful and elegant experiments showed that epithelial population ‘basal A’ progenitor cells (Ep-CAM^-/low^/CD49f^+^), ‘luminal B’ progenitor cells (Ep-CAM^high^/CD49f^+^), and ‘luminal differentiated C’ cells (Ep-CAM^high^/CD49f^-^) differ in their ability to form mammospheres and colonies (A>B, while C had no ability) (Figure 1). Although all three populations are found in normal tissue, there is in tumor tissue a shift towards type C and a great decrease in type A, and the majority of the nine BC cell lines analyzed mostly exhibited a population B/C phenotype.

In general, CD44^high^/CD24^low^ cell surface markers were the most efficient panel for selecting normal epithelial progenitors. Further fractionation of CD44^high^/CD24^low^ cells may select for luminal progenitors within Ep-CAM^high^/CD49f^+^ cell types, and for basal progenitors within Ep-CAM^- or low^/CD49f^+^.

Primary BC tissues (mainly luminal Ep-CAM^high^) were found to contain CD44^high^/CD24^low^ cells in both CD49f^-^ and CD49f^+^ cancer cell fractions. Ghebeh *et al.* showed for the first time that the CD44^high^/CD24^low^ subpopulation within CD49f^high^ cell types had the highest efficiency compared with other well-known subpopulations (based on MUC-1^-^, ALDH^+^, and CD10^+^ expression).

From a cancer biology point of view, Ghebeh *et al.* have performed an interesting and comprehensive study comparing various subpopulations of cells with stem cell-like properties, supporting the notion that BCSC were predominantly CD49f^+^, and proposing the use of CD44^high^/CD24^low^ in combination with Ep-CAM/CD49f as valuable biomarkers to identify BC cells with enhanced mammosphere-forming and colony-forming ability.

### What do the CD44^+^/CD24^-/low^ and Ep-CAM^+^/CD49f^+^ biomarker combinations really tell us about the biology of breast cancer and the heterogeneity of cancer stem cells?

The phenotype of the normal human mammary gland stem/progenitor cells has been previously described in various reports as ALDH^high^, CD10^+^, CD44^high^/CD24^low^ or Ep-CAM^+^/MUC1^-^ and CD49f^+^[18].

Ghebeh *et al.* found that human mammary epithelial cells with a CD44^high^/CD24^low^ phenotype had the highest progenitor ability, providing a convincing demonstration that, in both normal and malignant breasts, there are multiple CD44^high^/CD24^low^ subpopulations. Within the basal Ep-CAM^-/low^/CD49f^+^ cells, the subpopulation of CD44^high^/CD24^low^ has the highest progenitor ability, whereas CD10^-^ cells have the lowest progenitor ability (that is, the lowest number of differentiated myoepithelial cells).

It is known that luminal mammary epithelial cells have a estrogen receptor-positive (ER^+^) cell population, whereas proliferating normal luminal cells are known to be ER^-^[1]. Interestingly, Ghebeh *et al*. showed a correlation between the CD44^high^/CD24^low^ phenotype and ER^-^ profile in normal mammary epithelial cells within Ep-CAM^high^/CD49f^+^, in full agreement with the progenitor ability of these cells.

As depicted in Figure 1, there is a clear drift in patients with BC towards population C (Ep-CAM^high^/CD49f^-^) which almost doubled, whereas population A (Ep-CAM^-/low^/CD49f^+^) dramatically decreased in BC compared with healthy tissue.

For the first time, it has been shown that the majority of BCSC with CD44^high^/CD24^low^ phenotype exist mainly in the Ep-CAM^high^/CD49f^+^ fraction of cancer cells, revealing a significant difference in CD44^high^/CD24^low^ expression (in Ep-CAM^high^ BC cells) between ER and basal subtypes of BC in CD49f^+^ cancer cells only [21]. In other words, putting together the pieces of the puzzle, these data imply that CD49f (α-6 integrin molecule), if used in combination with CD44^high^/CD24^low^ markers, may be able to link the stem/progenitor cell profile with the heterogeneity of BC subtypes. Thus, BCSCs can best be enriched by selecting for tumor cells with the CD44^high^/CD24^low^/ALDH^high^ phenotypes within Ep-CAM^high^/CD49f^+^ BC cells.

### Conclusions and future perspectives

Improving technological methods, such as single-cell analysis [23] for earlier detection and diagnosis of human BC, in conjunction with the discovery and validation of powerful combinations of BCSC biomarkers, may represent key tools to obtain a significant reduction in morbidity and mortality in BC.

Analysis of the BC microenvironment [6,7,23] and the novel identification of pure/specific epithelial stem/progenitor cells [21], will allow detection of alterations within biochemical, morphological, and molecular pathways promoting cancer initiation, progression, invasion, and metastasis, taking into account the different stem/non-stem cell compositions and interactions in the human breast microenvironment [11,17,18].

Therefore, although confirmatory studies are needed, it is time to move on to the new paradigm highlighted by Ghebeh *et al.*, namely, that the majority of BC cells have a luminal Ep-CAM^high^ phenotype with a very small percentage of cancer cells of the Ep-CAM^-/low^/CD49f^+^ phenotype.

Further research is required, which should focus on single-cell omic approaches, with particular attention on basal Ep-CAM^low^ primary cancer cells, as these may correspond to the mammary stem cell-enriched population in the normal breast gland.

Finally, the recently published study in *BMC Cancer* on CD44^high^/CD24^low^/CD49f^+^ biomarkers represents a shining example of how the combination of more biomolecules (singularly not perfectly accurate) may significantly improve and strengthen the measurement of BCSCs with significantly higher stem/progenitor ability. These experiments suggest that these biomarkers will be a useful BC biomarker panel and the best phenotype to identify human BCSCs and to better understand BC biology.

Future developments in onco-single-cell-omics [23] will potentially revolutionize cancer biology and clinical practice, providing better understanding of BC heterogeneity, how BCSCs evolve, and which BC cells to target in order to avoid drug resistance [18].

## Abbreviations

ALDH: Aldehyde dehydrogenase; BC: Breast cancer; BCSC: Breast cancer stem cell; CSC: Cancer stem cell; ER: Estrogen receptor.

## Competing interests

The author declares that has no competing interests.

## Authors’ information

FM holds a professional position as Aggregate Professor of Cell Biology at the University ‘Carlo Bo’ of Urbino, care of the Dept of Biomolecular Sciences. He has held the position of the Chief Investigator of Grant Awards on Intraductal Approach to Breast Cancer Research, funded by DSLRF (Santa Monica, CA, USA) since 2005, and has been President of the Association of Fight Against Cancer of Urbino (AULCT-ONLUS), Italy since 2009.

## Pre-publication history

The pre-publication history for this paper can be accessed here:

http://www.biomedcentral.com/1741-7015/11/169/prepub
